# A promising small molecule binding pocket in class B GPCRs: expanding potential for drug development

**DOI:** 10.1038/s41392-023-01598-y

**Published:** 2023-08-11

**Authors:** Huan Xiao, Qian Sun, Qiu Sun

**Affiliations:** 1 Department of Biotherapy, Cancer Center and State Key Laboratory of Biotherapy, West China Hospital, Sichuan University/West China School of Nursing, Sichuan University, Chengdu, 610041 China; 2 West China Medical Publishers, West China Hospital, Sichuan University, Chengdu, 610041 China

**Keywords:** Structural biology, Molecular medicine

Recently, Xu et al.^1^ have published in *Nature*, solved the high-resolution structure of a small molecule agonist PCO371 and Gs bound human parathyroid hormone receptor 1 (PTH1R) by cryo-electron microscopy. This study reveals a novel conserved small molecule agonist binding pocket of class B G protein-coupled receptors (GPCRs) and provides new insights into drug development for various therapeutic indications.

GPCRs are a family of receptors with a seven transmembrane (7TM) domains that mediate various downstream signals by activating G proteins or recruiting β-arrestins. As the largest family of cell membrane receptors encoded by the human genome, GPCRs are widely involved in human physiological and pathological processes and have become the most important therapeutic targets. Class B GPCRs are main peptide hormone receptors, response to endocrine or paracrine peptide hormones, and consist of 15 receptors including glucagon receptor (GCGR), glucagon-like peptide-1 receptor (GLP-1R), gastric inhibitory polypeptide receptor (GIPR) and parathyroid hormone receptor 1 (PTH1R). PTH1R regulates calcium homeostasis and bone development through the activation of parathyroid hormone (PTH) and PTH-related peptide (PTHrP) and is a target for existing drugs that treat osteoporosis. Although some high-affinity peptide drugs have been successfully marketed, including GLP-1 analogs for type 2 diabetes and PTH or PTHrP analogs for osteoporosis, they have many disadvantages such as inconvenient subcutaneous injection, expensive, and associated with side effects such as headache and nausea.^2^ Unlike injectable drugs, small-molecule drugs have better drug properties and easier for oral transmucosal delivery. However, due to the complex structure and signaling pathways of class B GPCRs, the development of small-molecule drugs targeting receptors has always been urgent and challenging. At present, the small molecule drug development mainly focuses on GLP1R and PTH1R. PCO371 is a potent oral PTH1R small molecule agonist which is currently being studied in Phase 1 clinical trials in hypoparathyroidism. However, the molecular mechanism by which PCO371 activates PTH1R remains unclear.^3^

**Fig. 1:**
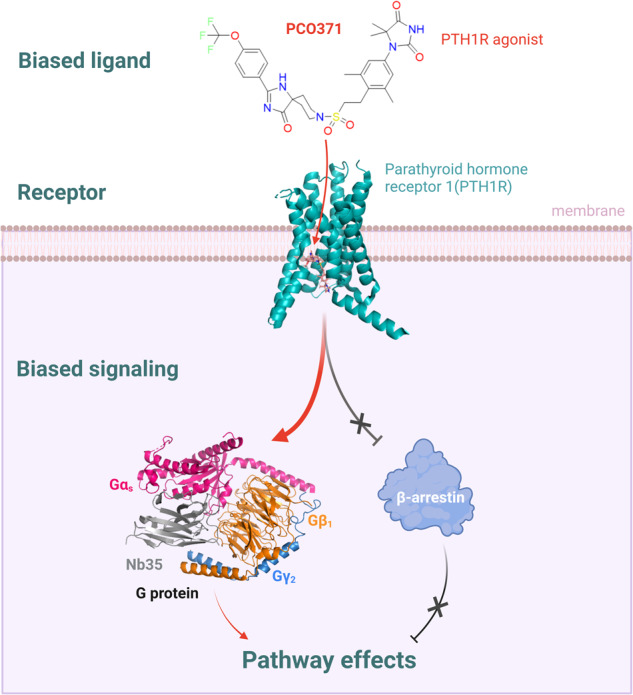
Structure basis of PTH1R activation by a biased intracellular agonist. PCO371-PTH1R-Gs-complex (PDB:8JR9)

It is well-known that GPCRs signal through multiple transducers, including G proteins, GPCR kinases (GRKs), and β-arrestins. While these signaling pathways can be selectively activated in a ‘biased’ manner. Drugs that selectively activate (i.e., biased agonists) one of the GPCR pathways tend to have better efficacy and fewer side effects than conventional agonists that simultaneously activate both GPCR signaling pathways. The design and synthesis of biased molecules is an inevitable trend in the development of GPCR targeting drugs. So far, the majority of biased agonists have been discovered through accidental screening, and the mechanism of action is unknown.^4^ Therefore, it is of great scientific importance to understand the mechanism of biased signaling mediated by biased agonists for biased drug discovery targeting GPCRs (Fig. 1).

In the study, the structure of a ternary complex of PCO371-PTH1R-Gs with a high resolution of 2.57 Å was obtained through cryo-electron microscopy. Structural analysis and functional experiments revealed that PCO371 binding pocket is different from the endogenous ligand binding sites, and biases to activate the G protein signaling pathways rather than β-arrestin signaling pathways. The PCO371 binding pocket consists of intracellular portion of TM2, TM3, TM6, and TM7, as well as helix 8. PCO371 binds to the cytoplasmic interface of PTH1R and Gs protein, adopts a horizontal U-shape posture, and wraps around the lower half of TM6. Completely different from the previously reported binding sites for ligands in all GPCRs, the conserved PxxG motif in the middle of TM6 (P415^6.47b^-L416^6.48b^-F417^6.49b^-G418^6.50b^) in PTH1R moves outward to form the binding pocket of PCO371. The authors found that P415^6.47b^ is a key residue for PCO371 selectivity. Furthermore, based on receptor sequence alignment and homology modeling, it is suggested that the amino acid residues located in the PCO371 binding pocket are highly conserved in class B GPCRs, which is proved by cAMP accumulation assay. It is enabling PCO371 to activate eight receptors in class B GPCRs and allows other receptors to respond to PCO371 with one- or two-residue mutations. Most importantly, this biased agonist helps to reduce the side effects caused by the β-arrestin signaling pathway and improve the safety and efficacy of drugs.

In summary, the structure of the PCO371-PTH1R-Gs complex provides the structural basis for small molecule agonist binding and activation of PTH1R, revealing a novel binding mode of PCO371, and finding that PCO37 has a biased activation of the G protein signaling pathway. This study provides new insights and avenues for small-molecule drug design and development of class B GPCRs. By targeting this conserved pocket, it is possible to develop drugs that are more selective and have fewer side effects than current treatments. It is worth mentioning that another study of Xu’s research group^5^ reported the first high-resolution structure of Neurotensin receptor 1 (NTSR1) and GRK2 complex in *Nature* on August 2. This study provides a structural explanation for the molecular mechanism of the NTSR1 biased agonist SBI-553. It shows that the binding mode of SBI-553 is compatible with the binding of β-arrestins, but conflicts with the binding of Gq protein, resulting in a biased downstream signal. Together, these two studies provide the first comprehensive and systematic detailed molecular mechanism of receptor-biased signaling mediated by two different classes of biased agonists, which greatly advances our understanding of GPCRs biased signaling. It also provides a solid structural basis for the future development of biased drug targeting GPCRs.
